# How Do We Measure Social Management in Non-profit Organizations? A Scale Design Based on the Once Case

**DOI:** 10.3389/fpsyg.2021.652663

**Published:** 2021-07-29

**Authors:** Antonio Luis Moreno-Albarracín, Cristina Ortega-Rodríguez, José Carlos Álvarez-López, Pedro Núñez-Cacho

**Affiliations:** ^1^Department of Financial Economics and Accounting, University of Jaén, Jaén, Spain; ^2^Department of Business Organization, University of Jaén, Jaén, Spain

**Keywords:** management accounting, non-profit organizations, management indicators, Spanish National Organization for the Spanish Blind, credibility, management control

## Abstract

One of the most important current challenges facing non-profit organizations (henceforth, NPOs) is to demonstrate that resources are being used properly to fulfill their missions. The development of control mechanisms to facilitate the measurement of social goal fulfillment has thus become a priority. In this context, transparency and good governance are configured as essential strategic elements to build trust with different stakeholders. In this work, we show the value provided by management indicators as they have become a necessary tool to confirm that the use of resources, internal processes and decisions within NPOs are carried out with the highest levels of efficiency and excellence. Only in this way can social credibility be achieved. The success of an NPO is inextricably linked to the support of donors, users, public administration and society as a whole. To achieve our research objective, we build a measurement scale based on the case of the Spanish National Organization for the Blind (ONCE), one of the largest Spanish NPOs. Based on ONCE’s experience, we propose a management indicator model that covers all social dimensions. The model is empirically validated to standardize the indicators for the ONCE and for serving as a reference for other entities.

## Introduction

In recent decades, we have witnessed the strong development of non-profit organizations (NPOs), with the NPO becoming a unique model in the provision of essential social services for the community (Connolly et al., 2013; Amagoh, 2015). The high capacity for anticipation among NPOs has allowed them to implement management models that enable them to immediately address societal demands with efficient actions and procedures. In our current context, the increasing relevance of these entities is evident (Connolly et al., 2013), and their importance will continue to grow (Pennerstorfer and Rutherford, 2019) as social needs increase. The altruistic work of these organizations has received unquestionable social recognition (Rodríguez et al., 2012), mainly because these entities address a large part of the most important needs of our society (Schatteman, 2013).

The recent appearance of entities that engage in actions that hover around the border between legality and illegality (McDonnell and Rutherford, 2019) as well as vague legal frameworks have prompted debate regarding disclosure practices and accountability procedures among different interest groups (Hofmann and McSwain, 2013). There are significant differences in what members of an organization do and what they say they do that are related how they understand responsibility (Tacon et al., 2017). Transparency has become a key issue for NPOs (Sanzo-Pérez et al., 2017). The image they project to society has become essential (Tremblay-Boire and Prakash, 2015). The future of this entire sector will be conditioned by the confidence it is capable of projecting. The absence of transparency involves the loss of support from donors, volunteers, users and society in general, highlighting the failure of the organization (Hale, 2013).

Due to all of the above, there is a growing interest in the study of the governance and reporting of these organizations (Boomsma and O’Dwyer, 2019). Good governance and transparency become necessary for achieving credibility with stakeholders. In NPOs, the goodwill and altruism by which the actions of its members are governed cannot become an excuse for not carrying out optimal management. There is a need to measure actions in a systemic way to generate legitimacy and credibility (Arena et al., 2015). Society has delegated a responsibility to the non-profit sector, and resources must be effectively managed. Thus, it is necessary to show society that there are management systems that, supported by the highest levels of transparency, allow the achievement of the mission, i.e., the ultimate reason for which the entity was born.

The distinguishing features of NPOs are that they do not pursue a profitable purpose and that their ultimate objective is to provide an essential service for society, among others; these features as well as the difficulty of valuing intangibles that do not have a reference price, result in a unique situation regarding the supply of information. Financial measures are limited to understanding the performance of NPOs (Kim, 2017). The application of techniques and management methods from the business sector is not the most appropriate strategy and must in any case be preceded by adaptations that take into account the differences between the two environments (Urionabarrenechea et al., 2015).

We intend to analyse the economic management models currently used by NPOs, particularly by the Spanish National Organization for the Blind (ONCE^1^) as the selected entity of study, asking ourselves the following questions:

**RQ 1:** Are the current indicators of social management in NPOs the most appropriate for the assessment of their management of social services, in accordance with the requirements of transparency, good governance and excellence in the fulfillment of their missions?
**RQ 2:** Is it possible to develop a comprehensive set of management indicators to measure the social services of the ONCE, including the participation and validation of the institution itself?

Thus, the aim of this research is to design a measurement scale for social management, standardizing these indicators and providing them as a basis for other NPOs to support their achievement of their missions and increase their ability to convey confidence to interested groups.

This research article proceeds in several sections. In section “Theoretical Framework and State of the Issue,” we review the relevant literature on the conceptualization of the organizations that make up the third sector and we justify how management indicators turn into a tool that responds to information needs. In section “Empirical Study to Develop a Measurement Scale of Social Management,” we present an empirical study to develop a measurement scale of social management for the ONCE, a Spanish NPO. The findings are discussed in section “Discussion”. The last section “Conclusion” presents our main conclusions.

## Theoretical Framework and State of the Issue

### The Unique World of NPOs and Their Information Needs

Non-profit organizations are included within the so-called third sector, which is distinguished from the commercial sector by its social functions. The solidarity component makes the third sector unique, with profit maximization being irrelevant (Hofmann and McSwain, 2013) and the main objective being the achievement of a social mission (Gilchrist and Simnett, 2019). Surplus funds or residual income generated through NPO activity are dedicated to additional activities by the entity itself (Thorne and Venable, 2008). However, different authors (Teasdale, 2012; Salamon and Sokolowski, 2016; Coraggio, 2017) consider this unique characteristic to be limited in defining the conceptualization of NPOs, which is currently subject to a deep debate (Lorenzo et al., 2017).

Organizations that share aims and behaviors focused on a sense of altruism coexist in this sector alongside other organizations with much more disparate objectives and actions. In addition, the third sector is quite varied, and there is no consensus as to how it should be defined or classified (Baur and Schmitz, 2012; Amagoh, 2015). The sector is composed of a diversity of institutions (Salamon and Sokolowski, 2016) such as small local entities or large global organizations that operate in more than 50 countries with several million partners (Servós, 2007). A clear and understandable conceptualization remains one of the urgent needs in third sector studies (Salamon and Sokolowski, 2016).

In recent decades, we have witnessed an explosion of NPOs (Connolly et al., 2013). The development of the sector has been driven by multiple economic, social, political, and religious factors (Austin, 2000; Fernández and Gil, 2011; Cestari et al., 2018), becoming a key element to help meet the needs of society (Cabedo et al., 2018).

This growth introduces new opportunities, but at the same time, it imposes new needs, demands, and challenges that must be faced; while the measurement of performance in NPOs has received greater academic and professional attention, somewhat limited consideration has been dedicated to the design of measurement systems for these organizations (Moxham, 2009). Rey-Garcia et al. (2017) stated, “in the last decade, numerous efforts have been made to obtain more information on these evaluation practices.”

The very dynamics of NPOs, as well as the performance of their tasks within a framework of changing needs that require immediate attention, has led to their growth in a heterogeneous and unique environment (Olmeda, 2012). This environment is characterized by dispersed legal regulation, which is a field of incipient study in the accounting, economic and management fields. Thus, Anthony and Young (1988) and, more recently, Moreno et al. (2016) noted the contextual factors that differentiate NPOs and that affect their management control processes of their management, including the peculiarities of the developed activity, the raising of public funds, the favorable tax regime in which they operate, their political influences and their service orientation.

Stakeholder theory has multiple applications including business ethics, social responsibility, corporate governance and finance (Miles, 2017). This theory has its origin in sociology, organizational behavior, special interest policy and managerial self-interest (Gibson, 2000). The performance of an NPO is analyzed by numerous parties with different interests (Shea, 2012); hence, NPOs must respond to multiple stakeholders (Costa and Goulart da Silva, 2019). Therefore, the NPO must exercise responsibility by focusing its attention on how to respond to the diverse or conflicting expectations and demands of stakeholders and how to manage the organization to fulfill its mission and maintain its institutional legitimacy (Jeong and Kearns, 2015). This demand for responsibility makes the issue a pressing concern for both academics and non-profit professionals (Lecy et al., 2012).

### A Social Management Scale as a Tool of Good Governance in Non-profit Organizations

We consider stakeholder theory to be particularly valuable since there is research that shows that the probability that an NPO is perceived as effective increases when the NPO manages to align the various expectations of each group regarding good governance (Wellens and Jegers, 2014). In this way, a discussion arises about what is considered good governance in these organizations (Willems et al., 2012; Byers et al., 2015).

Huetos (2008) introduced the so-called paradox of non-profit entities: these entities receive large amounts of resources based on the confidence that they will be applied to the intended outcomes, but on the other hand, as a consequence of the mirage guaranteeing the altruism and goodness of those who manage NPOs, some entities are less accountable than others. An essential aspect for donation decisions is the ability of external stakeholders to access specific information about the operations of an NPO (Behn et al., 2010).

The research on social management measurement scale indicators within NPOs is in an initial phase, with few studies carried out to date (Connolly et al., 2013; Righi and Andreoni, 2014; see for example: Arena et al., 2015; Rey-Garcia et al., 2017). These studies have not resolved the existing information deficit, and the implementation of new particularly flexible models, whose adaptation to the peculiarities of each organization does not become an obstacle for the organization, is considered a “challenge for the future” (Codorniu, 2009). The investigation of these organizations and the environment in which they operate is in an initial stage that, according to Moura et al. (2015), for now can benefit only from work that is more exploratory and attempts to better define the characteristics of NPOs that are important for the implementation and use of performance measurement systems.

Aspects related to trust, decision-making instruments and transparency in accountability play a priority role in the management of NPOs (Weidenbaum, 2009; Keating and Thrandardottir, 2017).

On the other hand, it is not possible to apply indicators of profit or profitability obtained to NPOs since it is necessary to take into account different social objectives that, in most cases, are difficult to quantify. In short, all the measures that are commonly used in the business environment are meaningless in the non-profit sector, and instead, other elements must be designed to assess the achievements of NPOs, such as indicators (Perdomo, 2007).

For this reason, it is necessary to identify another way to measure whether NPO management is adequate, for which it is undoubtedly essential to know whether the resources the NPO has are allocated to the intended purpose (González-Sánchez and Rúa-Alonso, 2007), thus fulfilling the expectations that the stakeholders have about the organization.

Based on the above, we state the following two hypotheses sequentially, so the second hypothesis will be tested in the event that the first hypothesis is confirmed:

**Hypothesis 1:** NPOs require systems for evaluating the management of social services under the current economic conditions characterized by greater competition and demands.
**Hypothesis 2:** Efficiency, effectiveness and excellence are key criteria for the development of sets of indicators.

Therefore, we first specify the objectives of the research to analyse the economic-financial and budgetary control models of the ONCE. The analysis allows us to study and confirm, where appropriate, the information needs not covered by the ONCE’s management tools. Second, and mainly, we propose a model of management indicators covering all social dimensions, and we take into account previous initiatives in the same field, as well as the contributions of the entities themselves.

## Empirical Study to Develop a Measurement Scale of Social Management

### The Selection of the Entity and the Importance of the ONCE Case

We focus this study on the ONCE case, which is especially important since in Spain, the social economy has been configured as an unquestionable global reality, with a level of involvement even greater than that of other countries in the European environment. The social economy generates 10% of the gross domestic product (compared to 8% in the European Union and 7% worldwide), and it is made up of 42,140 entities of different sizes that operate in all economic sectors and provide 2.2 million direct and indirect jobs (compared to 13.6 million in the European Union) (CEPES, 2019). The predominance of this business model has made Spain a benchmark in terms of the institutional recognition of the social economy, as it is the first member state of the European Union to pass a social economy law (Law 5/2011, from 29 March) (Gobierno de España, 2011), that defines and legally recognizes this business model in accordance with its values and principles, making Spain a pioneer country in this field. Spain was also the first state in the European Union to have implemented a 2017–2020 Social Economy Strategy that is supported by 63 measures that, in turn, are based on 11 strategic axes.

The ONCE was chosen for this study because it is a particularly relevant entity in the field of service provision, not only because it is one of the organization with the greatest economic volume dedicated to social interests in Spain but also because it is completely different than the rest of the NPOs. ONCE has been able to develop an exclusive model of economic management, a system to garner resources and a procedure to provide unique services. Thus, the reasons for our interest in analyzing this entity are evident. The economic volume it generates; its unique sources of income, such as the sale of coupons; and its management model have allowed it to be considered by the Spanish state as the only “singular entity” (Law 5/2011, from 29 March).

Because ONCE is a single system, the need for analysis by outsiders who give legitimacy to the system used is more justified. In addition, ONCE has recently undergone an organizational change of profound complexity, which has generated high-value information needs that force us to rethink the study and development of management indicators.

At present, the organic-functional structure that covers the entire Spanish territory comprises 17 territorial delegations, 5 area directorates, 11 support directorates, and 5 autonomous centers. All these entities enjoy autonomy, as the delegations and directorates correspond to a geographical area, while the autonomous centers cover the entire country for specific tasks. Under the auspices of the General Council, there are three executive areas in which the activity of the ONCE Social Group is formed: The general management of the ONCE (income from gambling and the provision of social services competences), the ONCE Foundation (cooperation with other disabilities) and ILUNIÓN (social enterprises group). At the end of 2019, the ONCE Social Group presented the following data (Table 1).

**TABLE 1:** Detailed information on the ONCE Social Group.

Description	Magnitude
Income	3,338,000,000
Consolidated profit after tax	80,929,000
Assets volume	1,355,882,000
Workers	72,693 workers
	Workers with disabilities (58%) Workers without disabilities (42%)
Affiliated people (blind and partially sighted)	72,231 persons
Promoted employment for people with disabilities in third parties (2010–2019)	78,903 persons
SDGs alignment	Goals 4, 5, 7, 8, 9, 10, 11, 12, 13, 16, and 17

*Authors’ elaboration based on the “Shared value report. Aggregate executive summary of ONCE, ONCE Foundation and ILUNIÓN 2019” and “Annual Accounts”.*

Through initial interviews held with a large group of managers, the currently proposed model of indicators was examined, and a set of weaknesses was identified, especially in relation to the assessment of social management, which is considered a priority in this area. According to these managers, there is a need to design new management indicators that are applicable to each of the social activities carried out by the organization. This first analysis highlighted two points. First, ONCE has made various attempts in the past to design and use management indicators, but without achieving the objectives initially set. There is a standardized and homogeneous group of indicators that are widely applied to all management centers. Second, in the absence of a common standard, it is standard practice in a large number of centers for management indicators to be applied based on autonomously determined criteria. The data from the questionnaire (Supplementary Annex 3) that was subsequently conducted indicated that this practice occurred in 78.78% of centers. In addition, there are essential activities in the organization for which no indicators of any kind have been configured to evaluate the management carried out.

### Process of the Development of the Social Management Measurement Scale

#### Step 1: Analysis, Selection, and Classification of Starting Indicators and Dimensions

**FIGURE 1:**
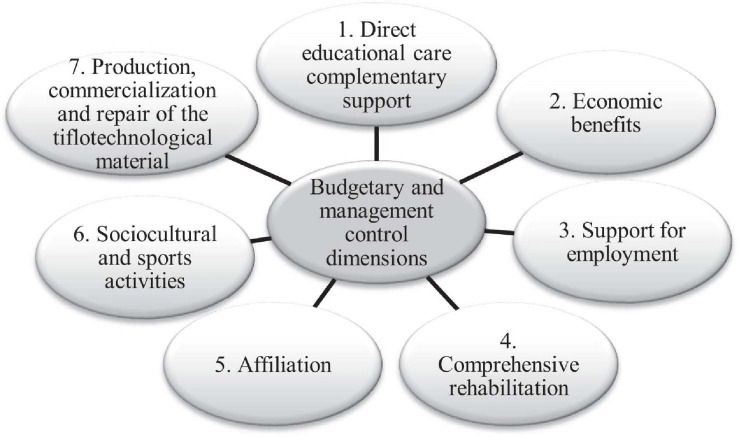
Social management measurement scale design process. Source: Authors.

**FIGURE 2:**
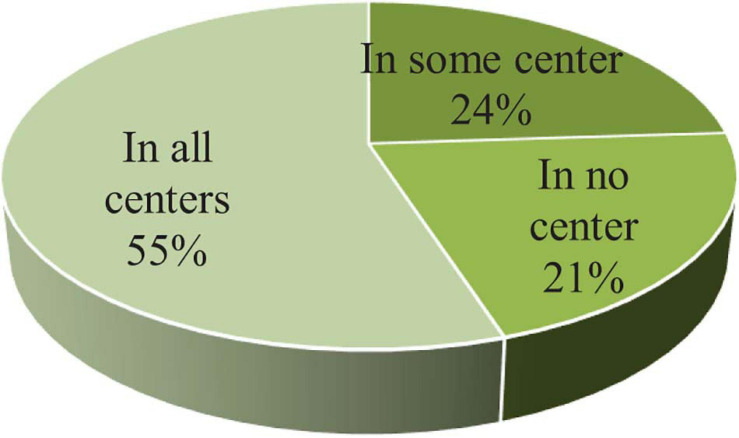
Budgetary and management control dimensions of ONCE. Source: Authors.

To develop this social management scale (see Figure 1), as a starting point, we used documents 3 and 26 of the Spanish Association of Accounting and Business Administration (AECA, hereafter) (AECA, 2002, 2012). These documents contains indicators of municipal management given that municipalities have certain similarities with NPOs. The similarities are based on the fact that the performance of municipalities is not aimed at achieving any financial benefit; therefore, creating a valuation model of the services provided presents difficulties. By analogy, we can apply the indicators for municipalities to NPOs. However, we are aware that the resulting system of indicators will always leave room for debate with regard to its composition and which of them can be used to measure a specific dimension (Valencia et al., 2015). Thus, the design and sources of data collection will vary depending on the aspects to be measured. After the aforementioned indicators were compiled, they were grouped according to their field of action, following the criteria applied by the entity itself, in the following dimensions (Figure 2) (for more details, see Supplementary Annex 1).

Content validity was determined by using 12 executive experts from the organization in which the case study was developed, from different geographic centers. The following criteria were followed for their election: (1) they had worked as managers in ONCE, (2) they had worked in other NPO’s. For the content validation of the scale, a cover letter was included explaining why the expert managers were invited to participate, along with clear and concise instructions on how to rate each item of those who made up the scale. This analysis addressed four fundamental points, first, the importance of each indicator in the scale; second, the clarity with which each indicator was written; third, how was each indicator necessary for the scale, and fourth, what suggestions could be made to improve each indicator. All this applying a 4-point Likert scale (being 1 nothing important; 2 something important; 3 quite important and 4 very important). Ratings of 1 and 2 were considered invalid content, while ratings of 3 and 4 were considered valid content (Wynd et al., 2003). To check the clarity of the measurement scale, a 3-point Likert scale was used (being 1 nothing clear; 2 needs some revision; 3, very clear).

#### Step 2: Grouping of Indicators According to Their Nature

After that, each group of indicators was subdivided then into the following typology (Galera, 1998; AECA, 2002, 2012): first, economic and input indicators, which relate the costs incurred with respect to another variable; second, effectiveness and output indicators, which indicate the entity’s ability to achieve its intended objectives; third, efficiency and process indicators, which relate to the results obtained regarding the resources consumed, which introduced difficulty in determining how to value the services provided; and fourth, excellence indicators, which were intended to measure the quality of the service provided.

#### Step 3: Application of the Questionnaire

To perform the investigation, the questionnaire was designed exclusively for managers and intermediate managers who assumed the highest-level decision-making tasks in each of the centers where these services are provided: territorial and zonal delegations. In addition, we opted to expand the population under study, seeking the opinion of other center directors who, although they are not specifically responsible for the comprehensive provision of services, did not have affiliates of their center. In terms of formal questions that we considered important to consider before beginning our analysis, we note the 108 proposed indicators (see Supplementary Annex 2).

The following variables were evaluated by the surveyed managers: usefulness of the indicators and their ease of implementation. Using a Likert-type scale, the response scales ranged from 1 (minimum or most negative value) to 5 (maximum or most positive value). The indicators were divided into seven major dimensions. Each dimension was subdivided into four typologies. Purposive sampling was performed, with a final sample size of 33 centers. The response rate was 78.57% (33 centers of a total of 42 centers to which the questionnaire had been sent). Since there were 108 indicators in total and 2 variables to be measured for each indicator (the utility and ease of implementation), we received a total of 216 complete responses.

#### Step 4: Descriptive Analysis

First, we describe to the actual situations of the centers that we verified based on the questionnaire responses. Subsequently, due to the peculiarities of ONCE, with exclusive access to management positions was given to blind or partially sighted people, we consider the opinion of these managers regarding the proposed indicators to be relevant, and we examine the extent to which the size of the center influenced their jobs. We continue by analyzing the managers’ assessments of each indicator of the proposed social management scale regarding the utility and ease of implementation, and we group the indicators by management dimension and typology. Finally, we focus on the process followed to validate the proposed scale.

**FIGURE 3:**
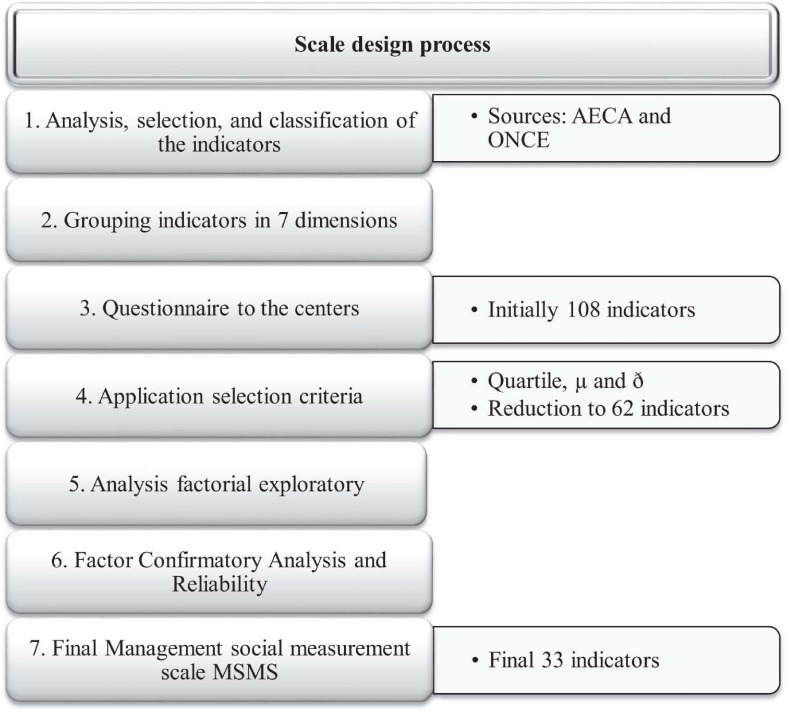
Analysis by centers: use of indicators in the proposed fields. Source: Authors.

A first question that guided the path of this research was centered on whether the different ONCE centers developed indicators with the objective of evaluating the management of social services. From the responses received, we infer that a majority of centers have developed their own systems of indicators that are independent and different from those of the other centers (Figure 3):

• A total of 54.55% of the centers (18) have designed indicators and use them regularly in all the fields analyzed.
• A total of 24.25% (8) have designed indicators and use them regularly in some, but not all, of the fields analyzed. Of these 8 centers, 75% of them (6) use indicators in most of the fields, while the remaining 25% do not (2 centers).
• A total of 21.20% of the centers (7) have not designed indicators and therefore are not employing indicators in any of the analyzed fields.

Therefore, 78.79% (26) have designed and used indicators regularly in all or some of the fields analyzed.

In the responses, it was observed that in 13 fields, certain centers did not use their indicators, and it was understood that there is a specific delegation of which tasks are measured (e.g., certain territorial delegations have a Centre of Educational Resources but do not consider educational indicators to be their responsibility). Apart from the above, on three occasions, no response was obtained. Considering all the responses regarding all the fields, indicators are used in 151 cases, while they are not used in 64 cases.

In addition, we tried to determine which centers use indicators to a greater or lesser extent in relation to the typology according to which they were divided. Starting from the type of center, we compared them based on the use indicators in most or all the fields versus in a minority of the fields or no field. It can be deduced that the centers with the highest hierarchical level in the territorial structure of the ONCE were those with the highest propensity to use indicators: territorial delegations, 92.3%; area directorates, 80%; and support directorates 55.5%.

#### Step 5: Analysis of Results and Selection of Indicators

Our goal is to analyse the results and to refine the scale from the information obtained. The questionnaire provided to the interviewees measured 2 attributes of each of the 108 indicators provided: the ease of implementation of the indicator in the organization and the usefulness of the indicator in the opinion of the interviewee. Subsequently, the results analysis process began. For this analysis, the arithmetic mean (μ), standard deviation (σ), and quartile (Q) were calculated for each indicator. Subsequently, three criteria were established to accept or reject an indicator based on the statistics described (see Table 2).

**TABLE 2:** Criteria for selection of indicators.

Rule	Decision		
1. Quartile of membership	a. If x_i_ = Q_1_ or Q_2_ or Q_3_		Accepted
	b. If x_i_ = Q_4_		Checked
2. Value of the standard deviation	a. If σ_i_ ≥ 1		Rejected
	b. If σ_i_ < 1		Checked
3. Value of the arithmetic mean	a. If μ_i_ ≤ 3.35		Rejected
	b. If μ_i_ > 3.35		Accepted

*Authors.*

After the analysis, the 81 indicators included in quartiles 1, 2, and 3 were incorporated into the scale. The remaining 27 indicators were analyzed, and it was found that in 8 of them, the standard deviation was greater than one; thus, they were rejected. With the remaining 19, the arithmetic mean was verified to be greater than 3.5. It was found that 11 of them did not meet this requirement; thus, they were rejected, and the remaining 8 were incorporated into the scale. After the first descriptive statistical analyses of the sample, the information on the scale included 89 indicators.

#### Step 6: Factorial Exploratory Analysis and Validation of the Proposed Social Management Scale

After applying the selection criteria, we proceeded to the statistical validation of the social management measurement scale. To do this, we applied two psychometric tests: first, an exploratory factor analysis (EFA) and, second, a Cronbach’s alpha test to verify reliability.

The dimensionality of the scale was analyzed using EFA. EFA is a multivariate analysis technique that seeks to reduce the number of items and identify the underlying structure of the analyzed data. Within the EFA, we used the principal component extraction method, which transforms the original variables into a new set of variables, which are called principal components. The analysis of the mean explained variance (AVE) was also used.

During the EFA, those variables whose weight in the dimension was very low were excluded. In this way, we managed to improve the variance explained by the dimension. Table 3 shows the number of items eliminated for each dimension.

**TABLE 3:** Items eliminated during the EFA of each dimension.

Dimension	Number of items eliminated
Educational care	2
Economic benefits	4
Employment support	6
Comprehensive rehabilitation	4
Affiliation	4
Sociocultural and sports activities	7
Production and comerc. tifl.	0
Total	27

*Authors.*

Finally, after the EFA, 27 indicators were eliminated; thus, the scale was made up of a total of 62 indicators. The results of the EFA are shown in Table 4.

**TABLE 4:** Exploratory factor analysis results of each dimension.

Dimension	Indicator	Weight	Average (%)	Dimension	Indicator	Weight	Averages (%)
Educational care	EDUCA1	0.774	65.80	Affiliation	AFFIL1	0.701	80.89
	EDUCA2	0.8			AFFIL2	0.562	
	EDUCA3	0.827			AFFIL3	0.83	
	EDUCA4	0.755			AFFIL4	0.757	
	EDUCA5	0.853			AFFIL5	0.473	
	EDUCA6	0.74		Sociocultural and sports activities	SOCIO1	326	52.30
	EDUCA7	0.764			SOCIO2	0.501	
	EDUCA8	0.895			SOCIO3	0.703	
	EDUCA9	0.72			SOCIO4	0.84	
	EDUCA10	0.864			SOCIO5	0.786	
	EDUCA11	0.906			SOCIO6	0.405	
Economic benefits	ECONOM1	0.745	53.89		SOCIO7	0.766	
	ECONOM2	0.654			SOCIO8	0.376	
	ECONOM3	0.691			SOCIO9	0.805	
	ECONOM4	0.611			SOCIO10	0.342	
	ECONOM5	0.857			SOCIO11	0.142	
	ECONOM6	0.822		Production and comerc. tifl.	PRODUC1	0.83	70.73
	ECONOM7	0.728			PRODUC2	0.663	
Employment support	EMPL0Y1	0.762	54.75		PRODUC3	0.504	
	EMPL0Y2	0.805			PRODUC4	0.687	
	EMPL0Y3	0.725			PRODUC5	0.848	
	EMPL0Y4	0.855			PRODUC6	0.333	
	EMPL0Y5	0.736			PRODUC7	0.837	
	EMPL0Y6	0.508			PRODUC8	0.686	
Comprehensive rehabilitation	REHAB1	0.528	52.02		PRODUC9	0.824	
	REHAB2	0.737			PRODUC10	0.792	
	REHAB3	0.314			PRODUC11	0.636	
	REHAB4	0.61			PRODUC12	0.67	
	REHAB5	0.746			PRODUC13	0.885	
	REHAB6	0.809					
	REHAB7	0.753					
	REHAB8	0.65					
	REHAB9	0.61					

*Authors.*

#### Step 7: Validation of the Scale: Reliability Criteria and Factor Confirmatory Analysis

The analysis of convergent and discriminant validity was proposed by Campbell and Fiske (1959) with the aim of establishing conceptual and empirical tests to determine construct validity. The procedure is based on the analysis of the correlations between variables. We say that there will be convergent validity when the phenomenon under study is corroborated by independent procedures. Based on the scale provided by the AFE, which reflected the measurement of Social Management in NPO’s. This consisted of seven dimensions and we proceeded to replicate its structure using confirmatory factor analysis (CFA) to check its validity. CFA is a technique based on the analysis of covariance structures, whose objective is to determine to what extent the scale that has been proposed in this work is consistent with reality.

To confirm that the theoretical model proposed by the proposed scale fits the data adequately, we have carried out evaluations using: χ^2^, the root mean square error of the Steiger–Lind approximation (RMSEA); the Bentler comparative fit index (CFI), the traditional goodness of fit index (GFI); and the MFI and IFI adjustment indices. The process was performed with the EQS software. Once it had been adjusted, it was evaluated. In all dimensions, successive re-specifications were necessary, eliminating those indicators that the parameter significance test, the residuals and the modification indices were advising. From these analyzes the measurement scale was formed. The results of the adjustment appear in Table 5.

**TABLE 5:** Fitting the each dimension models in the first order FCA.

	Fit indice							Reliability
Dimension	χ^2^	NFI	CFI	IFI	MFI	GFI	RMSEA	RHO
Educational care	9.320	0.958	0.980	0.980	0.991	0.985	0.05	0.72
Economic benefits	4.541	0.986	0.992	0.992	0.994	0.991	0.07	0.809
Employment support	0.18	0.989	0.995	0.995	0.997	0.993	0.05	0.796
Comprehensive rehabilitation	0.39	0.993	0.997	0.997	0.996	0.996	0.01	0.801
Affiliation	0.122	0.983	0.991	0.991	0.996	0.91	0.06	0.768
Sociocultural sports	0.002	0.925	0.930	0.930	0.949	0.951	0.05	0.826
Production and comerc. tifl.	92.4	0.917	0.934	0.934	0.865	0.919	0.06	0.9

*Authors.*

The evaluation of the indices shows a good fit when accepted criteria are used (McDonald and Ho, 2002; Kline, 2005). The review of the standardized residual matrix has not revealed anything that requires further modification. We also confirmed that all the model modification indicators were small; suggesting that the fit would not improve by incorporating more relationships into the model.

The convergent validity of the scale is verified from the results of the first order CFA carried out, where the coefficients λ, which measure the relationship between the observable and the latent variable, are all statistically significant at the 95% confidence level (*t* > 1.96) and exceed the value 0.5 in all cases (see Table 6).

**TABLE 6:** Reliability of the Cronbach’s α scale.

Dimension	Cronbach’s alpha	Number of items
Educational care	0.944	11
Economic benefits	0.832	7
Employment support	0.822	6
Comprehensive rehabilitation	0.868	9
affiliation	0.690	5
sociocultural and sports activities	0.898	11
Production and comerc. tifl.	0.958	13
Total scale	0.960	62

*Authors.*

Reliability is defined as the degree to which measurements are free from random errors. Thus, a scale or measuring instrument will be reliable when similar results are obtained by applying it two or more times to the same group of individuals. If the association between the variables that make up the scale is high, it will produce consistent results (Malhotra, 1997), and we can say that the scale is stable. This association is a necessary condition for the scale that is used to be valid (Peterson, 1994), and its calculation will indicate the quality of the instruments that is used, in the sense that the structure of the scale is correctly designed and, therefore, the measurements are free of deviations produced by causal errors (Camisón, 1999).

Regarding to the internal consistence of the Scale, the Cronbach’s α coefficient is one of the most widely used indicators to check both the reliability of the measurement instrument as a whole and that of each of its dimensions. In our study, we observed that the Cronbach’s α coefficient verifies what is recommended for exploratory studies (Table 7), and the composite reliability index is in line with that recommended by Bagozzi and Yi (1988). The final scales is showed in Table 8.

**TABLE 7:** Standardized solutions of FCA of social management NPO’s scale.

Factor	Indicator (33)	λ Coefficient	E_n_
			
Educational care	EDUCA1	0.523	0.852
	EDUCA2	0.821	0.57
	EDUCA3	0.461	0.888
	EDUCA4	0.484	0.875
	EDUCA5	0.584	0.812
Economic benefits	ECONOM1	0.528	0.849
	ECONOM2	0.514	0.858
	ECONOM3	0.909	0.416
	ECONOM4	0.816	0.579
Employment support	EMPLOY1	0.895	0.446
	EMPLOY2	0.587	0.81
	EMPLOY3	0.667	0.745
	EMPLOY4	0.651	0.759
Comprehensive rehabilitation	REHAB1	0.666	0.746
	REHAB2	0.49	0.872
	REHAB3	0.75	0.662
	REHAB	0.845	0.535
Affiliation	AFFIL1	0.529	0.849
	AFFIL2	0.397	0.918
	AFFIL3	0.712	0.702
	AFFIL4	0.903	0.43
Sociocultural and sports activities	SOCIO1	0.538	0.843
	SOCIO2	0.785	0.62
	SOCIO3	0.827	0.562
	SOCIO4	0.743	0.669
Production and comerc. tifl.	PRODUC1	0.661	0.75
	PRODUC2	0.828	0.561
	PRODUC3	0.821	0.57
	PRODUC4	0.707	0.707
	PRODUC5	0.877	0.481
	PRODUC6	0.74	0.673
	PRODUC7	0.44	0.898
	PRODUC8	0.69	0.723

*Authors.*

**TABLE 8:** Final scale.

Factor	Indicator	Description
Educational care	EDUCA1	Expenses of the educational attention dimension/budgeted expenses in said dimension
	EDUCA2	Number of students in the integrated teaching course/number of teachers
	EDUCA3	Number of users served in the dimension of educational attention/personnel expenses
	EDUCA4	Number of users served in the educational attention dimension/total costs
	EDUCA5	Number of care sessions received/number of school days
Economic benefits	ECONOM1	Number of users served in the dimension of economic benefits/unplanned number of users in that dimension
	ECONOM2	Expenses in the economic benefits dimension/budgeted expenses in that dimension
	ECONOM3	Number of users served in the dimension of economic benefits/personnel expenses
	ECONOM4	Number of users served in the dimension of economic benefits/total costs
Employment support	EMPLOY1	Total costs of employment support staff/number of people employed
	EMPLOY2	Expenses of the employment support dimension/budgeted expenses in that dimension
	EMPLOY3	Number of users served in the employment support dimension/number of people employed in this task
	EMPLOY4	Number of users served in the employment support dimension/total costs
Comprehensive rehabilitation	REHAB1	Total costs of the comprehensive rehabilitation service/number of affiliates of the center
	REHAB2	Number of users attended in the comprehensive rehabilitation service/number of users attended in said service
	REHAB3	Number of users attended in the comprehensive rehabilitation service/total costs
	REHAB	Number of workers employed in the comprehensive rehabilitation service/number of affiliates of the center
Affiliation	AFFIL1	Number of users attended in the affiliation to/number of people employed in this task
	AFFIL2	Number of claims in relation to the affiliation dimension/number of affiliation files processed in a year
	AFFIL3	Average number of days elapsed since membership is requested until it is resolved
	AFFIL4	Ratings of the survey to affiliate users
Sociocultural and sports activities	SOCIO1	Number of users served in the sociocultural and sports activities dimension/unplanned number of users in that dimension
	SOCIO2	Expenses for the sociocultural and sports activities dimension/budgeted expenses for that dimension
	SOCIO3	Number of users served in the dimension of sociocultural and sports activities/number of affiliates of the center
	SOCIO4	Number of users served in the dimension of sociocultural and sports activities/number of affiliates who request to participate in these activities
Production and comerc. tifl.	PRODUC1	Total costs of production, commercialization and repair of typhlotechnological material dimension/number of affiliates of the center
	PRODUC2	Number of users served in the production, commercialization and repair of typhlotechnological material dimension/planned number of users in that dimension
	PRODUC3	Number of users served in the production, commercialization and repair of typhlotechnology material dimension/number of affiliates of the center
	PRODUC4	Number of users served in the production, commercialization and repair of typhlotechnological material dimension/number of people employed in this task
	PRODUC5	customers attended in the production, commercialization and repair of typhlotechnological material dimension/total costs
	PRODUC6	Number of claims in relation to the production, marketing and repair of typhlotechnology material size/number of affiliates (in hundreds)
	PRODUC7	Evaluation of users in commercialization and repair of typhlotechnological material
	PRODUC8	Number of users of the activities in the production, commercialization, and repair of typhlotechnological material dimension/number of affiliates of the center

*Authors.*

## Discussion

The findings highlight the high value that is generally assigned to the proposed indicators, both in terms of their usefulness and their ease of implementation. Specifically, the mean value of the EASE variable is 3.9994, and its standard deviation is 0.2809, while the mean values of the UTILITY variable is 3.6638, and its standard deviation is 0.35626.

For 93 of the 108 proposed indicators, 86.11% of the cases, the ease of implementation is rated higher than the perceived utility, which can lead us to infer that, as long as the level of required utility exceeds a certain value that has been previously established, there should be no serious drawbacks to its incorporation. In the remaining 15 cases, the costs of the application of the indicators will have to be assessed, and any other difficulties that may indicated in the responses received will have to be explored. Of these 15 cases, 40% (6) belong to Dimension 1, “Educational care and complementary support,” while 3 indicators belong to Dimension 4, “Comprehensive rehabilitation,” and 2 belong to Dimension 3, “Support for employment”; finally, Dimensions 2 “Economic benefits,” 5 “Affiliation,” 6 “Sociocultural and sports activities,” and 7 “Production, commercialization and repair of the tiflotechnological material” each have 1 case.

The dimensions in which the implementation of indicators is considered easier are Dimensions 3 “Support for employment,” 4 “Comprehensive rehabilitation,” and 6 “Sociocultural and sports activities.” The dimensions for which implementation is considered more difficult are Dimension 7 “Production, commercialization and repair of the tiflotechnological material,” followed by Dimensions 1 “Educational care and complementary support” and 5 “Affiliation.” Certainly, the level of difficulty is relative since, as we noted, the dimension with the least ease of implementation is Dimension 7 “Production, commercialization and repair of the tiflotechnological material,” with a score of 3.82 points. Therefore, the indicators for Dimension 4 “Comprehensive rehabilitation” are the most valued in terms of their utility, followed by those of Dimensions 6 “Sociocultural and sports activities” and 1 “Educational care and complementary support.” By contrast, the indicators that are considered less useful are those in Dimensions 7 “Production, commercialization and repair of the tiflotechnological material,” 5 “Affiliation” and 2 “Economic benefits,” in this order.

**FIGURE 4:**
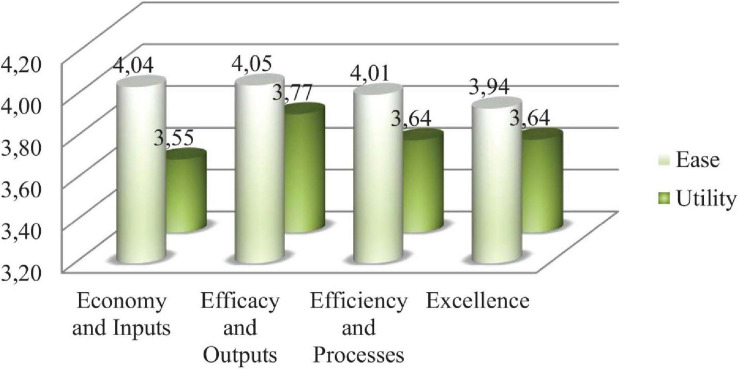
Averages by type of indicator. Source: Authors.

It is interesting to examine the aforementioned results based on the division of the set of indicators according to the four main economic typologies, as represented in Figure 4.

**FIGURE 5:**
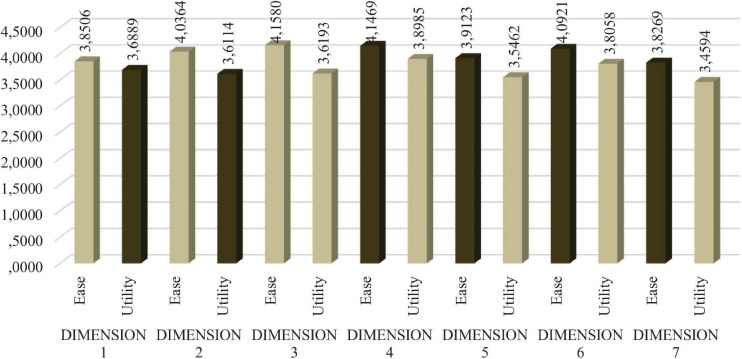
Ease/utility analyzed by dimensions. Source: Authors.

Segregating by indicator dimensions, we obtain the following representation of the mean values of each of the two attributes investigated (Figure 5).

We argue that the dimensions in which it is easier to implement indicators are number 3 “Support for employment,” followed by 4 “Comprehensive rehabilitation” and 6 “Sociocultural and sports activities.” The dimension that presents the greatest difficulty is 7 “Production, commercialization and repair of the tiflotechnological material,” followed by 1 “Direct educational care complementary support” and 5 “Affiliation.” It is true that this level of difficulty must be relativized since, as we noted, the least ease of implantation occurs in dimension 7, whose evaluation is 3.82 points.

In this case, we verify that the indicators in dimension 4 are the most valued in terms of usefulness, followed by 6 and 1. Conversely, those that are less useful are found in dimensions 7, 5 and 2 “Economic benefits,” in that order.

We can see that the effectiveness and output indicators are viewed as the easiest to implement and, at the same time, the most useful. The economy and input indicators are also considered easy to implement but not very useful, and finally, the efficiency and process indicators and excellence indicators are evaluated as having a high ease of implementation and being reasonably useful.

## Conclusion

One of the great challenges facing NPOs today is the need to demonstrate that the funds received are managed efficiently. In addition, it is imperative that NPOs demonstrate that such funds are mainly intended to fulfill the entity’s reason for being: its mission. For this reason, such organizations are obliged to develop internal management mechanisms that show the degree of fulfillment of their social objectives.

Sociological contributions have focused on the relationship of the NPO and its environment (Helmig et al., 2004) and thus, social management is a way by which the stakeholders and their collective voice can bring change and generate representativeness (Iwamoto et al., 2019). In this sense, stakeholders including donors and public administration, demand reliable information to conclude whether the resources are used for the entity’s social purpose.

This work has implications for the social management theory as this theory is understood as the process that allows social groups to influence decision-making processes of an organization. The goal of social management is to achieve favorable social order, equity, and justice and maintain social harmony (Shuzhuo et al., 2013). Four components are included in the “social management theory”: solving social problems, regularizing social behavior, coordinating social relations, and resolving social risks (Ding, 2011). We focus on the first component “solving social problems” as NPOs were created to attend social needs being its main objective the achievement of a social mission (Gilchrist and Simnett, 2019) addressing a wide range of issues for the public benefit (Balboa, 2014).

Therefore, measuring compliance with social management becomes a matter of high strategic value to generate social trust and provide a control tool to different social groups.

In this study, we defend the use of an adequate scale of measurement through indicators as one of the most interesting means to measure social management in the field of NPOs.

The study highlights that high value is generally assigned to the proposed indicators, both in terms of their usefulness and their ease of implementation. Specifically, the mean value of the EASE variable is 3.9994, and its standard deviation is 0.2809, while the mean value of the UTILITY variable is 3.6638 and its standard deviation is 0.35626.

Therefore, we note that when incorporating an indicator into the measurement scales, managers take into account two main criteria: the ease of implementation and perceived utility. However, the ease of implementation is the most determinant criterion in 86% of cases (93 indicators). In the remaining 14% of the cases studied (15 indicators), other criteria, such as the costs of implementation, are used. From these last 15 indicators, 6 correspond to Dimension 1 “Direct educational care complementary support,” while 3 indicators belong to Dimension 4 “Comprehensive rehabilitation,” and 2 belong to Dimension 3 “Support for employment.” Finally, Dimensions 2 “Economic benefits,” 5 “Affiliation,” 6 “Sociocultural and sports activities” and 7 “Production, commercialization and repair of the tiflotechnological material” have one indicator each.

The dimensions in which the implementation of the indicators are considered to be easier are Dimension 3 “Support for employment,” followed by Dimensions 4 “Comprehensive rehabilitation” and 6 “Sociocultural and sports activities.” The dimensions presenting greater difficulty include 7 “Production, commercialization and repair of the tiflotechnological material,” followed by 1 “Educational care and complementary support” and 5 “Affiliation.”

However, the differences between the levels of difficulty between dimensions is small, with the dimension with the least ease of implementation being Dimension 7 “Production, commercialization and repair of the tiflotechnological material,” with a score of 3.82 points.

“Effectiveness and Output” indicators are both the easiest to implement and the most useful. “Economy and Input” indicators are also easy to implement but are not perceived to be as useful as the previous indicators. Finally, “Efficiency and Process” indicators and “Excellence” indicators have great ease of implementation and are perceived as reasonably useful.

Hypothesis 1 was formulated with reference to the fact that NPOs need a system for evaluating the management of social services under the current economic conditions characterized by greater competition and demands; this hypothesis was confirmed.

Hypothesis 2 was confirmed since a comprehensive set of management indicators has been developed to measure the ONCE’s social services. The instrument has great potential for practical application because it was designed based on information from the organization. The indicators have been incorporated from the dimensions of efficiency, effectiveness, and excellence, with the aim of standardizing them and offering them as a basis for the measurement of social management in other NPOs.

As limitations of the research, we point out those that are derived from having studied a specific case. For the application of this measurement scale to other social organizations, an adaptation should be made taking into account the size and field of action of each entity. This question provides a possible line of future research, together with the possibility of expanding the research to relevant organizations in other countries.

## Data Availability Statement

The original contributions presented in the study are included in the article/Supplementary Material, further inquiries can be directed to the corresponding author/s.

## Author Contributions

All authors contributed equally to the conceptualization, the methodology, formal analysis and for the writing, review, and editing final version of the manuscript.

## Conflict of Interest

The authors declare that the research was conducted in the absence of any commercial or financial relationships that could be construed as a potential conflict of interest.

## Publisher’s Note

All claims expressed in this article are solely those of the authors and do not necessarily represent those of their affiliated organizations, or those of the publisher, the editors and the reviewers. Any product that may be evaluated in this article, or claim that may be made by its manufacturer, is not guaranteed or endorsed by the publisher.

## Footnotes

1 Organización Nacional de Ciegos Españoles.

## Supplementary Material

Supplementary Annex 1 Budgetary and management control dimensions of once.

Supplementary Annex 2 Battery of indicators proposed for standardization.

Supplementary Annex 3 Questionnaire.
