# Supplementary material for: How Do We Measure Social Management in Non-profit Organizations? A Scale Design Based on the Once Case
Source: Front Psychol. 2021 Jul 29;12:652663. doi: 10.3389/fpsyg.2021.652663 (PMC8359739; doi:10.3389/fpsyg.2021.652663)
Supplement: Supplementary Annex 1 — Budgetary and management control dimensions of once. [file Data_Sheet_1.pdf]

frontiers

Supplementary Material

1 ANNEX 1: BUDGETARY AND MANAGEMENT CONTROL DIMENSIONS OF ONCE.

No	NAME	DESCRIPTION
**Dimension 1**	Direct educational care complementary support	The provision of educational support and interventions to achieve personal development and comprehensive training for people with blindness or severe visual impairment.
**Dimension 2**	Economic benefits	The provision of different financial and material benefits, such as social welfare, personal autonomy, and education, for members in need.
**Dimension 3**	Support for employment	The integration and inclusion of job-seeking affiliates in the labour market, as well as continued support for those already integrated through the provision of various forms of information and orientation and the prevention of exclusionary actions.
**Dimension 4**	Comprehensive rehabilitation	Support for the achievement of maximum personal autonomy for persons in rehabilitation, attending to their needs, interests and capacities, as well as carrying out actions aimed at improving the accessibility of the environment of the members.
**Dimension 5**	Affiliation	The performance of actions inherent to the ONCE affiliation process and those related to the prevention of blindness and visual impairment, as well as collaboration with associations.
**Dimension 6**	Sociocultural and sports activities	The offering of activities with the aim of aim, on the one hand, occupying individuals' free time in a creative and educational way and increasing the cultural engagement of people with visual disabilities and, on the other hand, promoting the creative capacities, hobbies and interests of this sector.
**Dimension 7**	Production, commercialization and repair of the tiflotechnological material	The design, development and adaptation of articles that contribute to improving the quality of life of blind people or people with serious visual deficiency.

2 ANNEX 2: BATTERY OF INDICATORS PROPOSED FOR STANDARDIZATION:

I. INDICATORS OF DIRECT EDUCATIONAL CARE AND COMPLEMENTARY SUPPORT:

No.	ASPECT TO MEASURE	INDICATOR
A.- ECONOMY AND INPUTS		
1	Unit staff cost	- Total costs of educational care staff/No. of persons employed
2	Cost of the educational care dimension per affiliate	- Total costs of the educational care dimension/No. of affiliates of the centre
3	Cost of the educational care dimension per affiliate of school age	- Total costs of the educational care dimension/No. of affiliates of the centre in school age
B.- EFFECTIVENESS AND OUTPUTS		
4	Operational effectiveness	- No. of users served in the educational care dimension/No. of potential users in said dimension
5	Budgetary effectiveness in expenses	- Expenses of the educational care dimension/Budgeted expenses in said dimension
6	Overall effectiveness	- No. of users served in the educational care dimension/No. of affiliates of the centre
7	Specific effectiveness	- No. of users served in the educational care dimension/No. of school-age affiliates of the centre
C.- EFFICIENCY AND PROCESSES		
8	Actual staff efficiency	- No. of users served in the educational care dimension/No. of people employed in this task
9	Actual efficiency of teachers	- No. of students of the integrated teaching course/No. of teachers
10	Economic efficiency of staff	- No. of users served in the educational dimension dimension/Staff costs
11	Overall efficiency of the dimension	- No. of users served in the educational care dimension/Total costs
		D.- EXCELLENCE

No.	ASPECT TO MEASURE	INDICATOR
12	Human resources per school-age student	- No. of workers employed in the educational care dimension/No. of school-age affiliates of the centre
13	Volume of complaints or claims	- No. of claims in relation to the educational care dimension/No. of affiliates
14	Affiliate valuation	- Score obtained on surveys by the educational care dimension
15	Average delivery time of the dimension	- Average No of days elapsed from when the service is requested until it is provided to the member
16	Dimension intensity	- No. of attention sessions received/Nº of school days
17	School failure	- No. of suspended students/Total nº of students
18	Dropout rate	- No. students who drop out/total number of students

II. INDICATORS OF ECONOMIC BENEFITS:

No.	ASPECT TO MEASURE	INDICATOR
A.- ECONOMY AND INPUTS		
19	Unit staff cost	- Total costs of the staff of the economic benefits dimension/No. of persons employed
20	Cost of the economic benefits dimension per affiliate	- Total costs of the economic benefits dimension/No. of affiliates of the centre
B.- EFFECTIVENESS AND OUTPUTS		
21	Operational effectiveness of customer service	- No. of users served in the economic benefits dimension/Planned no. of users in said dimension
22	Operational effectiveness of processed benefits	- No. of services processed/No. of requests foreseen
23	Budgetary effectiveness in expenses	- Expenses of the economic benefits dimension/Budgeted expenses in said dimension
24	Overall effectiveness	- No. of users served in the economic benefits dimension/No. of affiliates of the centre

No.	ASPECT TO MEASURE	INDICATOR
C.- EFFICIENCY AND PROCESSES		
25	Actual staff efficiency	- No. of users served in the economic benefits dimension/No. of people employed in this task
26	Economic efficiency of staff	- No. of users served in the economic benefits dimension/Staff costs
27	Overall efficiency of the dimension	- No. of users served in the economic benefits dimension/Total costs
D.- EXCELLENCE		
28	Human resources per affiliate	- No. of workers employed in the economic benefits dimension/No. of affiliates of the centre
29	Volume of complaints or claims	- No. of claims in relation to the economic benefits dimension/No. of affiliates
30	Affiliate valuation	- Score obtained on surveys by the economic benefits dimension
31	Average time for granting the benefit	- Average no. of days elapsed from the request for the benefit until it is resolved
32	Average time for payment of the benefit	- Average no. of days elapsed from the date the benefit is granted until the member is paid

III. INDICATORS OF EMPLOYMENT SUPPORT:

No.	ASPECT TO MEASURE	INDICATOR
A.- ECONOMY AND INPUTS		
33	Unit staff cost	- Total costs of employment support staff/No. of persons employed
34	Cost of the employment support dimension per affiliate	- Total costs of the employment support dimension/No. of affiliates of the centre
B.- EFFECTIVENESS AND OUTPUTS		
35	Operational effectiveness	- Number of users

No.	ASPECT TO MEASURE	INDICATOR
36	Budgetary effectiveness in expenses	- Expenses of the employment support dimension/Budgeted expenses in said dimension
37	Overall effectiveness	- No. of users served in the employment support dimension/No. of affiliates of the centre
38	Specific effectiveness	- No. of users served in the employment support dimension/No. of affiliates of the centre who seek employment
C.- EFFICIENCY AND PROCESSES		
39	Actual staff efficiency	- No. of users served in the employment support dimension/No. of people employed in this task
40	Economic efficiency of staff	- No. of users served in the employment support dimension/Staff costs
41	Overall efficiency of the dimension	- No. of users served in the employment support dimension/Total costs
D.- EXCELLENCE		
42	Human resources per affiliate	- No. of workers employed in the employment support dimension/No. of affiliates of the centre
43	Volume of complaints or claims	- No. of claims in relation to the employment support dimension/No. of affiliates
44	Affiliate valuation	- Survey scores of the employment support dimension
45	Average delay time in obtaining employment	- Average no. of days elapsed from the moment the individual accesses the employment support dimension until he or she finds a job
46	Average time for granting employment benefits	- Average no. of days elapsed from the request for the benefit until it is resolved
47	Average time for payment of the benefit	- Average no. of days elapsed from the date the benefit is granted until the member is paid

IV. COMPREHENSIVE REHABILITATION INDICATORS:

No.	ASPECT TO MEASURE	INDICATOR
A.- ECONOMY AND INPUTS		
48	Unit staff cost	- Total costs of comprehensive rehabilitation staff/No. of people employed
49	Cost of the comprehensive rehabilitation service per affiliate	- Total costs of the comprehensive rehabilitation service/No. of affiliates of the centre
B.- EFFECTIVENESS AND OUTPUTS		
50	Operational effectiveness	- No. of users served in the comprehensive rehabilitation service/No. of users provided for in said service
51	Budgetary effectiveness in expenses	- Expenses of the comprehensive rehabilitation service/Costs budgeted in said service
52	Overall effectiveness	- No. of users served in the comprehensive rehabilitation service/No. of affiliates of the centre
53	Specific effectiveness	- No. of users served in the comprehensive rehabilitation service/No. of affiliates of the centre who request said service
		C.- EFFICIENCY AND PROCESSES
54	Actual staff efficiency	- No. of users served in the comprehensive rehabilitation service/No. of people employed in this task
55	Economic efficiency of staff	- No. of users served in the comprehensive rehabilitation service/Staff costs
56	Overall efficiency of the service	- No. of users served in the comprehensive rehabilitation service/Total costs
D.- EXCELLENCE		
57	Human resources per affiliate	- No. of workers employed in the comprehensive rehabilitation service/No. of affiliates of the centre
58	Volume of complaints or claims	- No. of claims in relation to the comprehensive rehabilitation service/No. of affiliates
59	Affiliate valuation	- Survey scores of the comprehensive rehabilitation service dimension

No.	ASPECT TO MEASURE	INDICATOR
60	Average time of delay in care	- Average no. of days elapsed from the request for the comprehensive rehabilitation service until the care begins

V. AFFILIATION INDICATORS:

No.	ASPECT TO MEASURE	INDICATOR
A.- ECONOMY AND INPUTS		
61	Unit staff cost	- Total costs of affiliation staff/No. of persons employed
62	Cost of the affiliation dimension per affiliate	- Total costs of the affiliation dimension/No. of affiliates of the centre
B.- EFFECTIVENESS AND OUTPUTS		
63	Operational effectiveness	- No. of users served in the affiliation dimension/Planned no. of users in said dimension
64	Budgetary effectiveness in expenses	- Expenses of the affiliation dimension/Budgeted expenses in said dimension
65	Overall effectiveness	- No. of users served in the affiliation dimension/No. of affiliates of the centre
66	Specific effectiveness	- No. of users served in the affiliation dimension/No. of affiliation requests
C.- EFFICIENCY AND PROCESSES		
67	Actual staff efficiency	- No. of users served in the affiliation dimension/No. of people employed in this task
68	Economic efficiency of staff	- No. of users served in the affiliation dimension/Staff costs
69	Overall efficiency of the dimension	- No. of users served in the affiliation dimension/Total costs
D.- EXCELLENCE		
70	Human resources per affiliate	- No. of workers employed in the affiliation dimension/No. of affiliates of the centre

No.	ASPECT TO MEASURE	INDICATOR
71	Volume of complaints or claims	- No. of claims in relation to the affiliation dimension/No. of affiliation files processed in a year
72	Affiliate valuation	- Survey scores of the affiliation dimension
73	Average time of delay in care	- Average no. of days elapsed from when the affiliation is requested until it is resolved

VI. INDICATORS OF SOCIOCULTURAL AND SPORTS ACTIVITIES:

No.	ASPECT TO MEASURE	INDICATOR
A.- ECONOMY AND INPUTS		
74	Unit staff cost	- Total costs of sociocultural and sports activities staff/No. of people employed
75	Cost of the sociocultural and sports activities dimension per affiliate	- Total costs of the sociocultural and sports activities dimension/No. of affiliates of the centre
B.- EFFECTIVENESS AND OUTPUTS		
76	Operational efficiency	- No. of users served in the sociocultural and sports activities dimension/Planned no. of users in said dimension
77	Efficiency compliance activities	- No. of activities carried out/No. of activities scheduled
78	Efficacy in places where the activities are offered	- No. of places where activity X is offered/Planned number of places where activity X is offered
79	Budgetary effectiveness in expenses	- Expenses of the sociocultural and sports activities dimension/Budgeted expenses in said dimension
80	General effectiveness	- No. of users served in the sociocultural and sports activities dimension/No. of affiliates of the centre

No.	ASPECT TO MEASURE	INDICATOR
81	Specific efficacy	- No. of users served in the sociocultural and sports activities dimension/No. of affiliates who request to participate in these activities
C.- EFFICIENCY AND PROCESSES		
82	Actual staff efficiency	- No. of users served in the sociocultural and sports activities dimension/No. of people employed in this task
83	Economic efficiency of staff	- No. of users served in the sociocultural and sports activities dimension/Staff costs
84	Overall efficiency of the dimension	- No. of users served in the sociocultural and sports activities dimension/Total costs
D.- EXCELLENCE		
85	Human resources per affiliate	- No. of workers employed in the sociocultural and sports activities dimension/No. of affiliates of the centre
86	Volume of complaints or claims	- No. of claims in relation to the sociocultural and sports activities dimension/No. of affiliates
87	Affiliate valuation	- Survey scores of the sociocultural and sports activities dimension
88	Actual users of the dimension	- No. of users of the activities in the sociocultural and sports activities dimension/No. of members of the centre
89	Variety of the offer in activities	Number of new activities this year / Number total activities organized in the previous year
90	Degree of coverage of activities	- No. of activities 100% covered/No. of activities organized
91	Affiliate proposals acceptance level	- No. activities organized/No. activities proposed by members
92	Degree of growth in activities	- No. of activities proposed in the current year/No. of activities proposed in the previous year

VII. INDICATORS OF THE PRODUCTION, COMMERCIALIZATION AND REPAIR OF TIFLOTECHNOLOGICAL MATERIAL:

No.	ASPECT TO MEASURE	INDICATOR
A.- ECONOMY AND INPUTS		
93	Unit staff cost	- Total costs of production, commercialization and repair of tiflotechnological material personnel/No. of persons employed
94	Cost of the production, commercialization and repair of tiflotechnological material dimension per affiliate	- Total costs of the production, commercialization and repair of tiflotechnological material dimension/No. of affiliates of the centre
B.- EFFECTIVENESS AND OUTPUTS		
95	Operational efficiency	- No. of users served in the production, commercialization and repair of tiflotechnological material dimension/Planned no. of users in said dimension
96	Budgetary effectiveness in expenses	- Expenses of the production, commercialization and repair of tiflotechnological material dimension/Budgeted expenses in said dimension
97	General effectiveness	- No. of users served in the production, commercialization and repair of tiflotechnological material dimension/No. of affiliates of the centre
C.- EFFICIENCY AND PROCESSES		
98	Actual staff efficiency	- No. of users served in the production, commercialization and repair of tiflotechnological material dimension/No. of people employed in this task
99	Economic efficiency of staff	- No. of users served in the production, commercialization and repair of tiflotechnological material dimension/Staff costs

No.	ASPECT TO MEASURE	INDICATOR
100	Overall efficiency of the dimension	- No. of users served in the production, commercialization and repair of tiflotechnological material dimension/Total costs
101	Economic efficiency of sales	- No. units sold/Total costs
102	Economic efficiency of repairs	- No. of repairs carried out/Total costs
D.- EXCELLENCE		
103	Human resources per affiliate	- No. of workers employed in the production, commercialization and repair of tiflotechnological material dimension/No. of affiliates of the centre
104	Volume of complaints or claims	- No. of claims in relation to the production, commercialization and repair of tiflotechnological material dimension/No. of affiliates (in hundreds)
105	Affiliate valuation	- Confidential survey scores of the production, commercialization and repair of tiflotechnological material dimension
106	Actual users of the dimension	- No. of users of the activities in the production, commercialization and repair of tiflotechnological material dimension/No. of affiliates of the centre
107	Units sold/units in stock	- No. of units sold/Average stock in exhibition store
108	Units repaired over total affiliates	- No. of units repaired/No. affiliates of the centre
