# Supplementary material for: How Do We Measure Social Management in Non-profit Organizations? A Scale Design Based on the Once Case
Source: Front Psychol. 2021 Jul 29;12:652663. doi: 10.3389/fpsyg.2021.652663 (PMC8359739; doi:10.3389/fpsyg.2021.652663)
Supplement: Supplementary Annex 3 — Questionnaire. [file Data_Sheet_2.pdf]

frontiers

Supplementary Material

ANNEX 3: QUESTIONNAIRE RELATING TO THE USE OF MANAGEMENT INDICATORS IN NON-PROFIT ORGANIZATIONS (NPOs).

ANALYZED NPO: ONCE

DIRECTEDD TO: TERRITORIAL DELEGATION, AREA DIRECTORATE and SUPPORT DIRECTORATE

(COMPLIANCE IS REQUESTED BY THE HEADS OF CENTER OR, WHERE CASE, THOSE WHO CONSIDER BETTER)

QUESTIONNAIRE:

1. THE CENTER IS:

   - ☐ TERRITORIAL DELEGATION
   - ☐ AREA DIRECTORATE
   - ☐ SUPPORT DIRECTORATE

2. POSITION WITHIN THE CENTER OF THE PERSON RESPONDING TO THIS SURVEY:
   - ☐ CENTER DIRECTOR
   - ☐ RESPONSIBLE FOR SOCIAL SERVICES FOR MEMBERS
   - ☐ OTHERS

3. PLEASE, INDICATE THE NUMBER OF YEARS YOU HAVE BEEN AS A MANAGER OR MIDDLE MANAGER AT ONCE: ______

4. NUMBER OF AFFILIATES ASSOCIATED WITH THE CENTER (In the case of an Area Directorate, those of the Support Directorates will be excluded. If it is a Territorial Delegation, those of the Area Directorates and those of the Support Directorates will be excluded):__________

5. NUMBER OF WORKERS AT THE CENTER (INCLUDING AGENCIES):
   - ➢ NON-SELLERS: ______
   - ➢ SELLERS: ______

6. NUMBER OF NON-SELLER WORKERS ASSOCIATED WITH THE SOCIAL AREAS DEPARTMENT:
   - ➢ ADMINISTRATIVES: ______
   - ➢ TECHNICIANS: ______

7. AMOUNT OF THE APPROVED EXPENDITURE BUDGET:
   - ➢ FUNCTION 2: _________________
   - ➢ TOTAL CENTER, INCLUDING FUNCTION 2:________________

8. IF YOU PREPARE ANY TYPE OF INDICATOR, WITH THE PURPOSE OF EVALUATING THE MANAGEMENT RELATED TO FUNCTION 2: WRITE THE INDICATOR AND ITS PURPOSE OF THE INDICATOR. PLEASE COMPLE THE TABLE WITH ALL THE MANAGEMENT INDICATORS THAT YOU USE, EVEN IF IT IS SPORADELY, FOR EACH OF THE AREAS INDICATED BELOW:

AREA	INDICATOR	PURPOSE
DIRECT EDUCATIONAL CARE COMPLEMENTARY SUPPORT		
ECONOMIC BENEFITS		
SUPPORT FOR EMPLOYMENT		
COMPREHENSIVE REHABILITATION		
AFFILIATION		
SOCIOCULTURAL AND SPORTS ACTIVITIES		
PRODUCTION, COMMERCIALIZATION AND REPAIR OF THE TIFLOTECHNOLOGICAL MATERIAL		

9. A BATTERY OF INDICATORS IS DETAILED BELLOW. THIS BATERY IS THE RESULT OF AN ANALYSIS ATHORS PERFORMED ON IMPROVEMENTS FOR THE MANAGEMENT PARTICULARIZED AT ONCE. ASSESS, ON A SCALE OF 1 TO 5, BOTH THE “UTILITY” THE INDICATOR COULD HAVE FOR THE MANAGEMENT, AS THE “EASE OF IMPLEMENTATION”.

   IN THE REFERRED SCALE, 5 IS THE MAXIMUM OR MOST POSITIVE VALUE (MAXIMUM PROFIT / MAXIMUM EASE OF IMPLEMENTATION) AND 1 IS THE MINIMUM OR MOST NEGATIVE VALUE (NULL UTILITY / NO EASE OF IMPLEMENTATION).

No.	INDICATOR	UTILITY					EASE OF IMPLEMENTATION				
		1	2	3	4	5	1	2	3	4	5
**I. INDICATORS OF DIRECT EDUCATIONAL CARE AND COMPLEMENTARY SUPPORT:**											
A.- ECONOMY AND INPUTS											
1	- Total costs of educational care staff/No. of persons employed										
2	- Total costs of the educational care dimension/No. of affiliates of the centre										
3	- Total costs of the educational care dimension/No. of affiliates of the centre in school age										
B.- EFFECTIVENESS AND OUTPUTS											
4	- No. of users served in the educational care dimension/No. of potential users in said dimension										
5	- Expenses of the educational care dimension/Budgeted expenses in said dimension										
6	- No. of users served in the educational care dimension/No. of affiliates of the centre										
7	- No. of users served in the educational care dimension/No. of school-age affiliates										
C.- EFFICIENCY AND PROCESSES											
8	- No. of users served in the educational care dimension/No. of people employed in this task										
9	- No. of students of the integrated teaching course/No. of teachers										
10	- No. of users served in the educational dimension dimension/Staff costs										
11	- No. of users served in the educational care dimension/Total costs										
D.- EXCELLENCE											
12	- No. of workers employed in the educational care dimension/No. of school-age affiliates										
13	- No. of claims in relation to the educational care dimension/No. of affiliates										
14	- Score obtained on surveys by the educational care dimension										
15	- Average No of days elapsed from when the service is requested until it is provided										
16	- No. of attention sessions received/Nº of school days										
17	- No. of suspended students/Total nº of students										
18	- No. students who drop out/total number of students										

No.	INDICATOR	UTILITY					EASE OF IMPLEMENTATION				
		1	2	3	4	5	1	2	3	4	5
**II. INDICATORS OF ECONOMIC BENEFITS:**											
A.- ECONOMY AND INPUTS											
19	- Total costs of the staff of the economic benefits dimension/No. of persons employed										
20	- Total costs of the economic benefits dimension/No. of affiliates of the centre										
B.- EFFECTIVENESS AND OUTPUTS											
21	- No. of users served in the economic benefits dimension/Planned no. of users in said dimension										
22	- No. of services processed/No. of requests foreseen										
23	- Expenses of the economic benefits dimension/Budgeted expenses in said dimension										
24	- No. of users served in the economic benefits dimension/No. of affiliates of the centre										
C.- EFFICIENCY AND PROCESSES											
25	- No. of users served in the economic benefits dimension/No. of people employed in this task										
26	- No. of users served in the economic benefits dimension/Staff costs										
27	- No. of users served in the economic benefits dimension/Total costs										
D.- EXCELLENCE											
28	- No. of workers employed in the economic benefits dimension/No. of affiliates of the centre										
29	- No. of claims in relation to the economic benefits dimension/No. of affiliates										
30	- Score obtained on surveys by the economic benefits dimension										
31	- Average no. of days elapsed from the request for the benefit until it is resolved										
32	- Average no. of days elapsed from the date the benefit is granted until the member is paid										

No.	INDICATOR	UTILITY					EASE OF IMPLEMENTATION				
		1	2	3	4	5	1	2	3	4	5
III. INDICATORS OF EMPLOYMENT SUPPORT:											
A.- ECONOMY AND INPUTS											
33	- Total costs of employment support staff/No. of persons employed										
34	- Total costs of the employment support dimension/No. of affiliates of the centre										
B.- EFFECTIVENESS AND OUTPUTS											
35	- Number of users										
36	- Expenses of the employment support dimension/Budgeted expenses in said dimension										
37	- No. of users served in the employment support dimension/No. of affiliates of the centre										
38	- No. of users served in the employment support dimension/No. of affiliates of the centre who seek employment										
C.- EFFICIENCY AND PROCESSES											
39	- No. of users served in the employment support dimension/No. of people employed in this task										
40	- No. of users served in the employment support dimension/Staff costs										
41	- No. of users served in the employment support dimension/Total costs										
D.- EXCELLENCE											
42	- No. of workers employed in the employment support dimension/No. of affiliates of the centre										
43	- No. of claims in relation to the employment support dimension/No. of affiliates										
44	- Survey scores of the employment support dimension										
45	- Average no. of days elapsed from the moment the individual accesses the employment support dimension until he or she finds a job										
46	- Average no. of days elapsed from the request for the benefit until it is resolved										
47	- Average no. of days elapsed from the date the benefit is granted until the member is paid										

No.	INDICATOR	UTILITY					EASE OF IMPLEMENTATION				
		1	2	3	4	5	1	2	3	4	5
IV. COMPREHENSIVE REHABILITATION INDICATORS:											
A.- ECONOMY AND INPUTS											
48	- Total costs of comprehensive rehabilitation staff/No. of people employed										
49	- Total costs of the comprehensive rehabilitation service/No. of affiliates of the centre										
B.- EFFECTIVENESS AND OUTPUTS											
50	- No. of users served in the comprehensive rehabilitation service/No. of users provided for in said service										
51	- Expenses of the comprehensive rehabilitation service/Costs budgeted in said service										
52	- No. of users served in the comprehensive rehabilitation service/No. of affiliates of the centre										
53	- No. of users served in the comprehensive rehabilitation service/No. of affiliates of the centre who request said service										
C.- EFFICIENCY AND PROCESSES											
54	- No. of users served in the comprehensive rehabilitation service/No. of people employed in this task										
55	- No. of users served in the comprehensive rehabilitation service/Staff costs										
56	- No. of users served in the comprehensive rehabilitation service/Total costs										
D.- EXCELLENCE											
57	- No. of workers employed in the comprehensive rehabilitation service/No. of affiliates of the centre										
58	- No. of claims in relation to the comprehensive rehabilitation service/No. of affiliates										
59	- Survey scores of the comprehensive rehabilitation service dimension										
60	- Average no. of days elapsed from the request for the comprehensive rehabilitation service until the care begins										

No.	INDICATOR	UTILITY					EASE OF IMPLEMENTATION				
		1	2	3	4	5	1	2	3	4	5
V. AFFILIATION INDICATORS:											
A.- ECONOMY AND INPUTS											
61	- Total costs of affiliation staff/No. of persons employed										
62	- Total costs of the affiliation dimension/No. of affiliates of the centre										
B.- EFFECTIVENESS AND OUTPUTS											
63	- No. of users served in the affiliation dimension/Planned no. of users in said dimension										
64	- Expenses of the affiliation dimension/Budgeted expenses in said dimension										
65	- No. of users served in the affiliation dimension/No. of affiliates of the centre										
66	- No. of users served in the affiliation dimension/No. of affiliation requests										
C.- EFFICIENCY AND PROCESSES											
67	- No. of users served in the affiliation dimension/No. of people employed in this task										
68	- No. of users served in the affiliation dimension/Staff costs										
69	- No. of users served in the affiliation dimension/Total costs										
D.- EXCELLENCE											
70	- No. of workers employed in the affiliation dimension/No. of affiliates of the centre										
71	- No. of claims in relation to the affiliation dimension/No. of affiliation files processed in a year										
72	- Survey scores of the affiliation dimension										
73	- Average no. of days elapsed from when the affiliation is requested until it is resolved										

No.	INDICATOR	UTILITY					EASE OF IMPLEMENTATION				
		1	2	3	4	5	1	2	3	4	5
VI. INDICATORS OF SOCIOCULTURAL AND SPORTS ACTIVITIES:											
A.- ECONOMY AND INPUTS											
74	- Total costs of sociocultural and sports activities staff/No. of people employed										
75	- Total costs of the sociocultural and sports activities dimension/No. of affiliates centre										
B.- EFFECTIVENESS AND OUTPUTS											
76	- No. of users served in the sociocultural and sports activities dimension/Planned no. of users in said dimension										
77	- No. of activities carried out/No. of activities scheduled										
78	- No. of places where activity X is offered/Planned No. of places where activity X is offered										
79	- Expenses sociocultural and sports activities dimension/Budgeted expenses dimension										
80	- No. of users served in the sociocultural and sports activities dimension/No. of affiliates										
81	- No. of users served in the sociocultural and sports activities dimension/No. of affiliates who request to participate in these activities										
C.- EFFICIENCY AND PROCESSES											
82	- No. of users served in the sociocultural and sports activities dimension/No. of people employed in this task										
83	- No. of users served in the sociocultural and sports activities dimension/Staff costs										
84	- No. of users served in the sociocultural and sports activities dimension/Total costs										
D.- EXCELLENCE											
85	- No. of workers employed in the sociocultural and sports activities dimension/No. of affiliates of the centre										
86	- No. of claims in relation to the sociocultural and sports activities dimension/No. affiliates										
87	- Survey scores of the sociocultural and sports activities dimension										
88	- No. of users of the activities in the sociocultural and sports activities dimension/No. members of the centre										
89	Number of new activities this year / Number total activities organized in the previous year										
90	- No. of activities 100% covered/No. of activities organized										
91	- No. activities organized/No. activities proposed by members										
92	- No. of activities proposed in the current year/No. activities proposed in the previous year										

No.	INDICATOR	UTILITY					EASE OF IMPLEMENTATION				
		1	2	3	4	5	1	2	3	4	5
A.- ECONOMY AND INPUTS											
93	- Total costs of production, commercialization and repair of tiflotechnological material personnel/No. of persons employed										
94	- Total costs of the production, commercialization and repair of tiflotechnological material dimension/No. of affiliates of the centre										
B.- EFFECTIVENESS AND OUTPUTS											
95	- No. of users served in the production, commercialization and repair of tiflotechnological material dimension/Planned no. of users in said dimension										
96	- Expenses of the production, commercialization and repair of tiflotechnological material dimension/Budgeted expenses in said dimension										
97	- No. of users served in the production, commercialization and repair of tiflotechnological material dimension/No. of affiliates of the centre										
C.- EFFICIENCY AND PROCESSES											
98	- No. of users served in the production, commercialization and repair of tiflotechnological material dimension/No. of people employed in this task										
99	- No. of users served in the production, commercialization and repair of tiflotechnological material dimension/Staff costs										
100	- No. of users served in the production, commercialization and repair of tiflotechnological material dimension/Total costs										
101	- No. units sold/Total costs										
102	- No. of repairs carried out/Total costs										
D.- EXCELLENCE											
103	- No. of workers employed in the production, commercialization and repair of tiflotechnological material dimension/No. of affiliates of the centre										
104	- No. of claims in relation to the production, commercialization and repair of tiflotechnological material dimension/No. of affiliates (in hundreds)										
105	- Confidential survey scores of the production, commercialization and repair of tiflotechnological material dimension										
106	- No. of users of the activities in the production, commercialization and repair of tiflotechnological material dimension/No. of affiliates of the centre										
107	- No. of units sold/Average stock in exhibition store										
108	- No. of units repaired/No. affiliates of the centre										
